# Dietary intake, forest foods, and anemia in Southwest Cameroon

**DOI:** 10.1371/journal.pone.0215281

**Published:** 2019-04-12

**Authors:** Caleb Yengo Tata, Amy Ickowitz, Bronwen Powell, Esi K. Colecraft

**Affiliations:** 1 Department of Nutrition and Food Science, University of Ghana, Accra, Ghana; 2 Forests, Resources and People, Limbe, Cameroon; 3 Center for International Forestry Research, Bogor, Indonesia; 4 Departments of Geography & African Studies, Pennsylvania State University, State College, Pennsylvania, United States of America; CSIR-Foood Research Institute, GHANA

## Abstract

**Background:**

Forest cover has been associated with higher dietary diversity and better diet quality in Africa. Anemia prevalence among women of reproductive age in sub-Saharan Africa is very high and diet is one known contributor of a high prevalence rate. We investigated whether living in communities with high forest cover was associated with better diet quality and lower anemia prevalence among women of reproductive age in Southwest Cameroon.

**Methodology:**

We conducted a cross-sectional survey of 247 women of reproductive age from four forest-based villages (n = 126) and four non-forest villages (n = 121). We assessed the Hemoglobin (Hb) levels, anthropometric status, and diet (by 24-hour recall), as well as anemia-related morbidity and socio-demographic characteristics. Differences between groups were assessed with Pearson’s chi-square and independent T-tests. We used a number of multivariate regression models to estimate the impacts of forest proximity on adjusted hemoglobin status of women of reproductive age, as well as to identify the most likely pathway through which forest proximity was important.

**Results:**

We found that women living in forest communities had higher adjusted hemoglobin levels (mean hemoglobin concentration 11.10±1.53 g/dl vs.10.68±1.55g/dl; p = 0.03 for women forest and non-forest communities respectively). Moderate to severe anemia prevalence was significantly higher in women living in the non-forest villages compared to women in forest villages (forest 63% vs. 73%; p = 0.04). Compared with women from non-forest villages, women from forest-based villages had consumed significantly more vitamin A rich fruits and vegetables and animal source foods, and more of these came from the forest (as opposed to the farm or purchased sources). We found that the consumption of *Gnetum africanum* (Eru), a leafy green vegetable that grows in forests of the Congo Basis, was best able to account for the higher levels of adjusted hemoglobin in women in forest communities.

**Conclusion:**

This study contributes to the growing evidence that in some circumstances, forests make important contributions to diet quality and nutrition. The results of this study suggest that plant foods from the forest may make important contributions to iron intake and reduce the risk of anemia in women. Efforts to prevent forest loss and maintain ecosystem services are warranted to enhance nutrition and health of forest-based communities.

## Introduction

While hunger has declined globally, rates of micronutrient deficiency have remained stubbornly high, especially in Africa [1, 2]. Iron deficiency anemia remains a major public health problem worldwide [1] and significantly increases the risk of maternal mortality, low birth weight and infant mortality [3–5]. Although global anemia prevalence declined between 1990 to 2010, nearly one-third (32.9%) of the world’s population is anemic, with women and children most affected and iron deficiency the main cause of anemia world-wide [2]. Countries in Africa have some of the highest prevalence of anemia [2, 5]. In Cameroon, the prevalence of anemia among women of reproductive age is 38.8%, higher for rural than urban women [6].

Iron-deficiency anemia can be the result of inadequate iron intake, as well as multiple physiological and genetic factors such as: age, pregnancy status, menstrual flow and hemolytic disease [6, 7]. Iron-deficiency anemia is also strongly associated with infection, most notably malaria, but also hook worm and schistosomiasis [7, 8]. One study suggested that over 25% of the cases of severe anemia in Sub-Saharan Africa were attributable to malaria [9].

**Fig 1:**
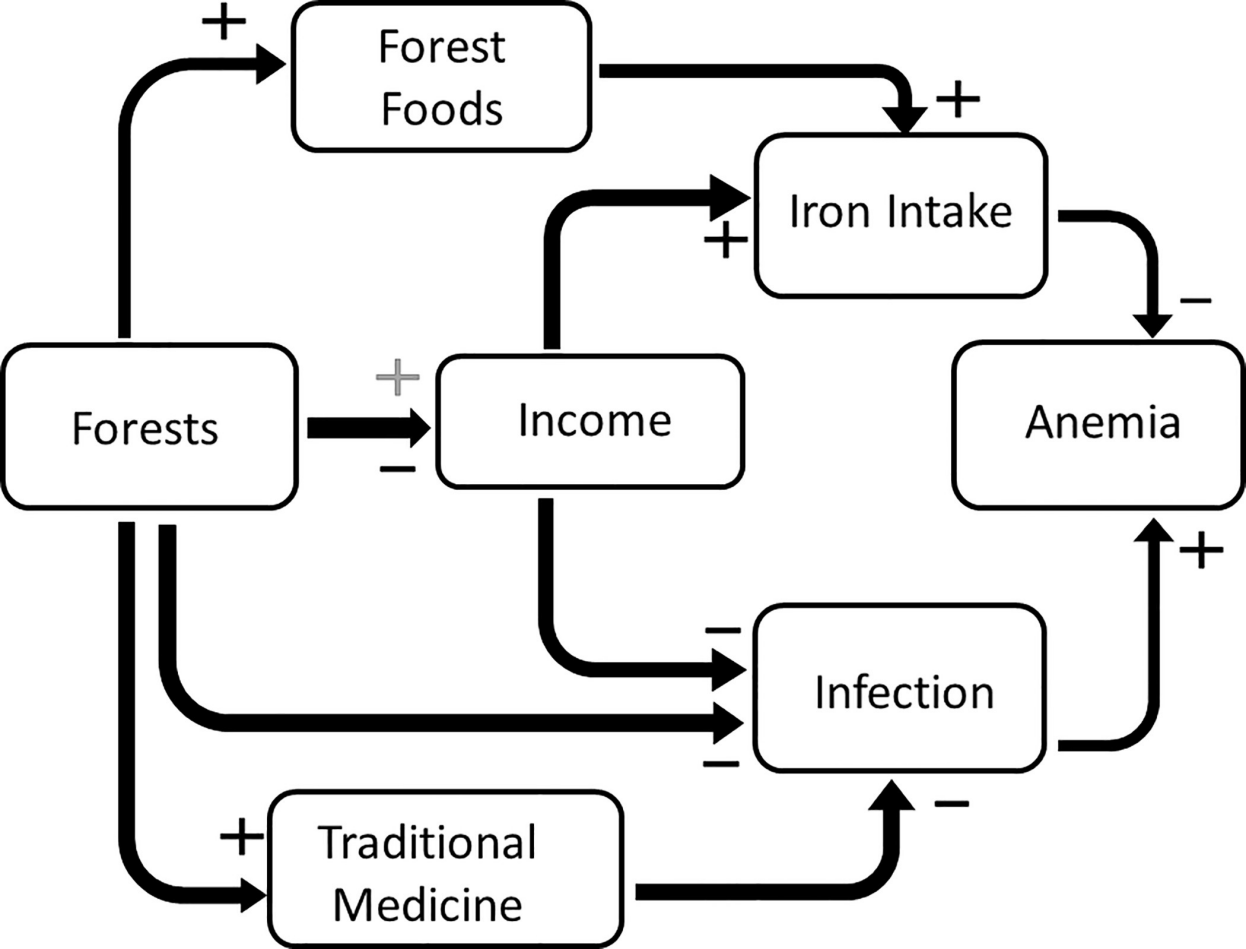
Potential pathways by which forest access might impact anemia. If forests are positively related to forest food use and such foods are rich in iron, the loss of forest may decrease forest food use, reducing iron intake and increasing anemia rates (which are negatively related to iron-rich food intake). Forests are posited as being negatively related to infection since there is some evidence that deforestation increases infection from vector borne diseases; and infection is positively related to anemia rates. Forests are also sources of traditional medicines, the loss of which could lead to an increase in infection. Finally, people living closer to forests may make income from the sale of forest goods or may have fewer economic opportunities due to less access to markets since forest areas typically have less infrastructure. Since income is positively related to intake of iron-rich foods and use on infection reducing medication, living near a forest could lead for fewer economic opportunities, less income and therefore lower consumption of purchased iron-rich foods and/or less access to medical care and higher infection rates; or if forest and income are positively related, the reverse will be the case.

Several socio-ecological and environmental factors can influence both iron intake and exposure to infection. In this paper we investigate whether living in close proximity to forests has an impact on iron-deficiency anemia, as well as the factors that might be responsible for such a relationship. Fig 1 presents some proposed pathways through which, change in forest proximity may impact hemoglobin and anemia status. Some studies have found that forest loss and fragmentation is associated with higher prevalence of malaria and other vector borne diseases [10–12], likely due to changes in habitat and breeding sites for malaria-carrying mosquitoes and other vectors [13]. Other research has shown an increase in hook worm and other parasitic infections following deforestation and other environmental changes [14, 15]. Froment et al. found that communities still living a traditional nomadic lifestyle in the forest in Cameroon had lower parasitic loads than those who were practicing agriculture in a forest environment [16]. Conversely, forest communities may consume more wild game (locally referred to as bushmeat) which may expose them to zoonotic parasitic infections [14, 17–19].

Forest proximity may also be related to anemia through differences in consumption of iron-rich foods. In areas where livestock raising is difficult, such as tropical forest areas where trypanosomiasis is endemic, bushmeat and fish often provide the majority of animal source foods consumed by local people [20, 21]. Past research has also demonstrated a relationship between forest cover and diet diversity in Africa [22–25]. Ickowitz et al. [22] reported a positive relationship between forest cover and dietary diversity using pooled demographic health survey (DHS) data from over 94,000 households across 21 African countries. The study also found a relationship between forest cover and fruit and vegetable consumption but not animal source food consumption [22]. Finally, if income levels are different in forest and non-forest communities, we might expect to see different levels of anemia, if income is spent either on iron-rich foods or on medical treatment and prevention for infections that increase the risk of anemia. People living in forest areas usually have lower incomes than those in other areas (possibly due to limited infrastructure and poor access to markets) and deforestation can be associated with increased income through the sale of agricultural products that are produced in the land cleared of what was previously forests [26]. Within forest areas, however, forest products can be important sources of income [26].

The current study is the first, to our knowledge, to test whether or not there is a relationship between forest proximity and anemia in women. We hypothesized that women living in high forest areas would have better access to iron-rich foods than their counterparts in the grassland areas, but that the impact of this on anemia could be modified by differences in the relative rates of infection across the two zones.

## Methodology

### Study site

**Fig 2:**
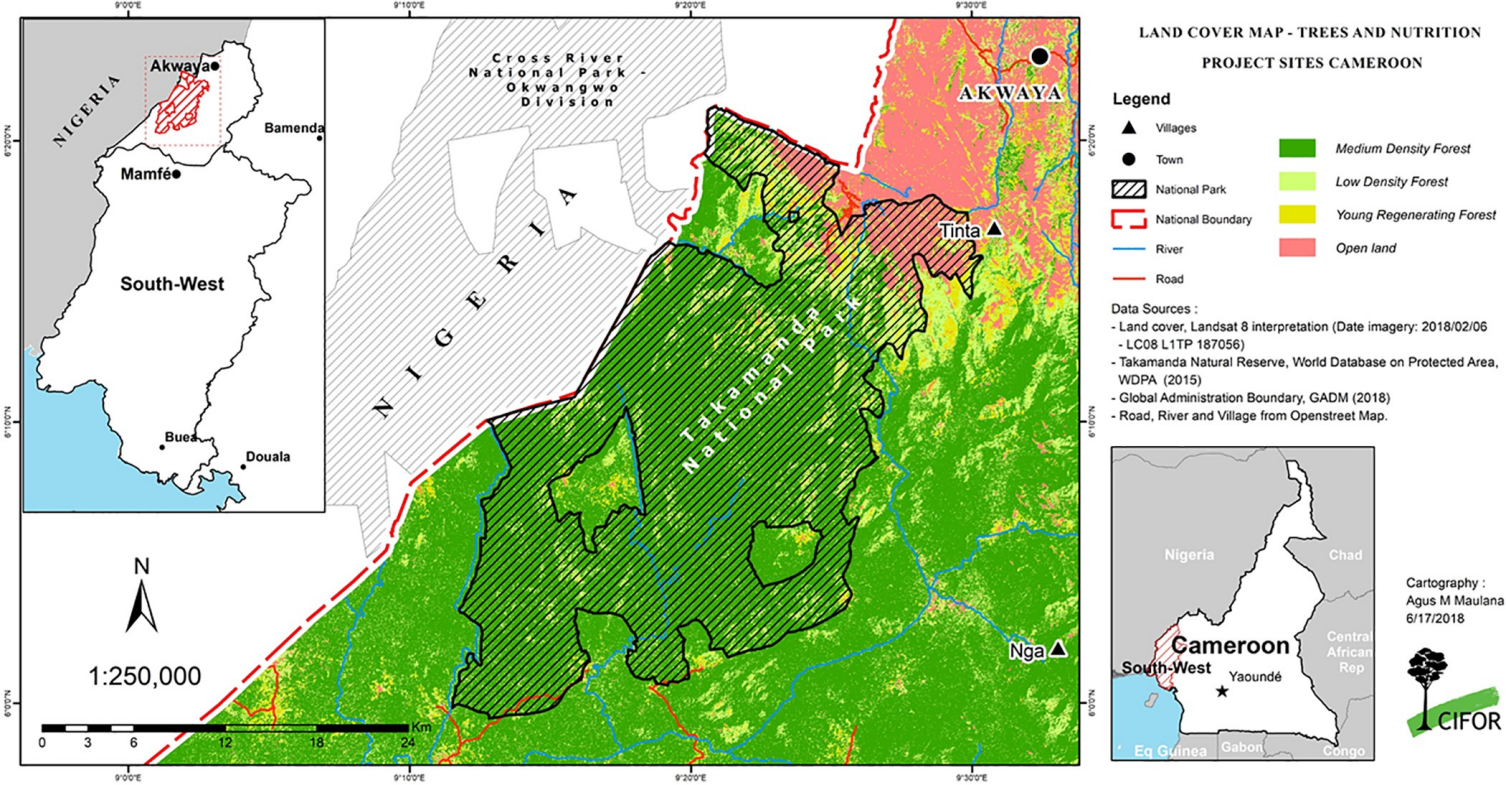
Map of study site.

The study site was located around the Takamanda rainforest region in Southwest, Cameroon (Fig 2). The area is ecologically diverse, with a naturally occurring mix of grassland/ savannah and humid tropical rainforest under varying degrees of protection. The area includes the Takamanda National Park, created in 2008 to form a trans-boundary protected area with Nigeria’s Cross River National Park to protect the remaining Cross River gorilla population. Before 2008, the area had been a Forest Reserve created by British colonial administration [27, 28]. The main ethnic groups in the area are the Anyangs, Bashos and the Becheves, all of whom practice a mix of livelihood activities including agriculture, hunting, fishing, and the gathering of non-timber forest products [28]. The main agricultural crops are maize, plantain, banana, yam, and cassava [29]. The region has two distinct seasons (rainy and dry) with most rainfall occurring from April to November, with a peak in July and a second peak in September [30]. The annual rainfall is about 4,500mm [31]. The roads in the area are mostly unpaved some of which are impassable during the rainy season. Access to most of the northern highland villages is generally by foot, but there has been a recent opening of some roads in the southern forest villages, increasing access.

This area was selected for a previous project (*Nutrition and Trees in sub-Saharan Africa* carried out by the Center for International Forestry Research) focusing on differences in diets between forest and non-forest communities. The area was chosen because it is one of the less developed areas of the country, with potentially high use of forest foods and because there are communities with high forest cover and low forest cover that are otherwise similar. The previous study classified villages as forest villages if they had more than 30% forest cover or non-forest villages if they had less than 30% forest cover. The study sites for the present research included eight of the villages from the original study: four from forest and four from non-forest areas chosen randomly from the 14 villages that were part of an earlier study.

### Study design and population

The study used a comparative cross-sectional design. The sample size for the study was estimated based on detecting a mean difference in Hb of 0.5g/dl between means with a two-sided 5% significance test and 80% power. The 2011 Cameroon DHS survey showed a difference of 0.5gm/dl in mean hemoglobin concentration between women living in different regions of the country [32], thus we took that as a reasonable benchmark for finding differences in two areas in our study. Data were collected from a subsample of women surveyed in an earlier project (*Nutrition and Trees in sub-Saharan Africa*) which ran from 2013–2015. A total of 247 women were randomly selected from the list of participants in the larger study (126 from the forest and 121 from the non-forest area). Participants were considered eligible if they were resident in the area for at least one year, were apparently healthy and willing to participate in the study.

### Data collection

The socio-demographic and dietary data from two seasons (July 2013, February 2014) from the larger study were used for this study. Additional data for the current study were collected in February 2016, including an additional 24 h recall, additional demographic information, as well as anthropometric and hemoglobin measurements for this subsample of women.

#### Questionnaire and 24 hour recall

A structured, pretested questionnaire was used to collect information on socio-economic characteristics, reproductive history, health seeking behavior and forest use. Questionnaires were administered in Pidgin English by trained enumerators at the homes of the participants. Women’s dietary intake was assessed using a 24hour recall where participants were asked to recall all foods and beverages, except water, they consumed in the past 24 hours. The 24-hour recalls were used to determine presence/absence of foods groups based on the FAO guidelines for measuring the minimum dietary diversity for women [33], as well as consumption of different nutritionally important foods. The number of days a woman consumed from each food group (or individual food for commonly consumed wild foods) over the three days of recall collected across three seasons was used in the analysis.

#### Anthropometric measurements

During the third round of data collection, a workstation was set up at a central location in each village (e.g. Chief’s house, community hall, or the primary school) for the anthropometric assessments. Women’s heights and weights were measured using standard procedures as described by WHO protocol [34]. Women’s weight was taken to the nearest 0.1 kg using a portable digital Tanita weighing scale and their heights were measured to the nearest 0.1 cm using a portable SECA^TM^ Stadiometer.

#### Biochemical measurements

Hemoglobin concentrations were determined on site using a battery operated portable HemoCue 201+ analyzer. The puncture site, on the middle finger, was cleaned and wiped dry using alcohol swap (70% alcohol) and then pressed lightly to stimulate the flow of blood. A lancet was used to prick the finger and the first drop of blood was wiped away and the next blood flow (10μl) was used for hemoglobin estimation. Values were recorded in grams per deciliters (g/dl). Women were classified as having anemia if they had a hemoglobin level <12g/dl or <11g/dl, for non-pregnant and pregnant women respectively. Results were given to each study participant. Women with severe anemia (hemoglobin less than 7g/dl for pregnant women or less than 9 g/dl for non-pregnant women) [35] were provided with a written referral form to the nearest community health center and counselled on foods to eat to help increase iron intake.

### Ethical considerations

This research was approved by the Ethics Committee of Basic and Applied Sciences University of Ghana (No: ECBAS 007/15-16) and the National Ethical Committee of Research for Human Health Cameroon (No: 2016/01/688/CE/CNERSH//SP). Community level approval and participation was also sought from village governments. Written informed consent (by signature or thumb print, if illiterate) was obtained from all participants after the study purpose and procedures had been explained. Women were given a tablet of soap as a token of appreciation for their time.

### Data analysis

Data entry was done using Microsoft excel 2010 and analyses were done using Statistical Package for Social Sciences (SPSS) version 20.0 and Statistical Analysis System (SAS). Regression analysis was done using Stata 15. The prevalence of anemia was calculated from the Hb results within each group. Independent t-tests were used to compare hemoglobin levels of both populations.

We ran regression models to investigate whether living in forest areas had an impact on anemia and adjusted hemoglobin levels, and then a set of several models to try to figure out the pathways. First, we ran two simple bivariate regressions of adjusted hemoglobin levels on a dummy variable indicating whether the woman lived in the forest or non-forest sub-sample and another using a dummy variable for anemia (moderate to severe; <8g/dl to 10.9g/dl) as the dependent variable against the forest dummy as the independent variable. Next, we ran six multiple regression models including different groups of control variables. We regressed adjusted hemoglobin levels on the forest dummy, but added women’s demographic and biological characteristics–we included controls for age, age squared (in case there is a different relationship for older women), education, and whether or not the woman was pregnant or menstruating at the time of the survey. In a second model, we included independent variables to control for two important causes of infection in the region–‘worms’ (a dummy equal to one if the woman reported having a worm infection in the last month) and ‘malaria’ (reported having malaria in the last month). Because these conditions were self-reported they are subject to error. The third model had controls for socio-economic and household characteristics including household size, whether or not the roof was made from aluminum (this likely reflects not only wealth, but also hygiene since there will likely be fewer rodents in the house), whether or not the household had a toilet, and a wealth index. The wealth index was calculated using principal components analysis [36] based on ownership of the following assets: radio, television, motorcycle, phone, and chainsaw.

The fourth and fifth models were used to try to isolate the impact of diet. Because hemoglobin levels are affected not only by what the individual ate the day before, but over longer periods of time, we used data on food consumption that were collected at three different points of time to try to capture household dietary patterns. In the fourth model, we regressed the adjusted hemoglobin level on forest and the average number of times the respondent consumed from the food groups commonly used in dietary studies: cereals; tubers; orange colored fruit (since these tend to be high in vitamin A); other fruits; dark green leafy vegetables (these tend to be high in vitamin A and iron); other vegetables; meat; dairy; fish; pulses, nuts and seeds; and oils and fats, across three seasons. In the fifth model, in addition to the food groups used in the previous model, we also included the most commonly eaten forest foods. Finally, in the last model, we included all of the independent variables in the same model. The last four models have fewer observations than the first three because they only included households who were interviewed in all three periods of data collection.

## Results

### Sociodemographic characteristics of women

The characteristics of the women who participated in the study are presented in Table 1. The mean age of the women was 29.7 ± 7.03 years. About 61% of the women were married and almost half (46%) of them did not have any formal education with significantly more of the women from the non-forest villages having no formal education (p<0.001). Farming was the main occupation for both groups of women. Women from forest-based communities were mostly of Anyang ethnicity while the non-forest village women were predominantly of Becheve ethnicity (note that these ethnic groups are very similar in terms of diet, livelihood and culture/ language). Mean number of births per woman (parity) was significantly higher for the forest women (5.1 ± 2.6 vs. 4.8 ± 2.3; p = 0.01) than non-forest women.

**Table 1:** Sociodemographic characteristics of women in the study.

Characteristic	Total (N = 247)	Forest (n = 126)	Non-forest (n = 121)	P-Value^1^
**Age (y)**	29.7±7.03^2^	28.8± 7.13	29.7±6.96	0.84
**Marital status**				
Single	60 (24.3)^3^	36 (28.6)	24 (19.9)	0.03
Married	151 (61.1)	74 (58.7)	77 (63.5)	
Cohabiting	36 (14.6)	16 (12.7)	20 (16.5)	
**Education**				
None	115 (46.6)	24 (19.0)	91 (75.2)	<0.01
Preschool	19 (7.7)	15 (11.9)	4 (3.3)	
Primary or more	113 (45.7)	87 (69.1)	26 (21.5)	
**Occupation**				
Farmer	224 (90.7)	113 (89.7)	111 (91.7)	0.01
Trader	11 (4.5)	2 (1.6)	9 (7.4)	
Other	12 (4.8)	11 (7.2)	1 (0.8)	
**Ethnicity**				
Anyang	101 (40.9)	94 (74.6)	7 (5.8)	<0.01
Becheve	113 (45.7)	0 (0)	113 (93.4)	
Basho and other	33 (26.2)	33 (26.2)	0 (0)	
**Religion**				
Christian	227 (91.9)	115 (91.3)	112 (92.6)	0.24
Traditional belief	18 (7.3)	11 (8.7)	7 (5.8)	
Other	2 (0.8)	0 (0)	2 (1.7)	
**Savings group**				
No, never	33 (13.4)	16 (12.7)	17 (14.0)	0.91
Yes in the past	26 (10.5)	14 (11.1)	12 (9.1)	
Yes, presently	118 (76.1)	96 (76.2)	92 (76.0)	
**Ever pregnant**				
Yes	242 (98)	123 (97.6)	119 (98.3)	0.68
No	5 (2)	3 (2.4)	2 (1.7)	
**Currently pregnant**				
Yes	25 (10.1)	11 (8.7)	14 (11.6)	0.45
No	222 (89.9)	115 (91.3)	107 (88.4)	
**Parity**	4.7±2.531	5.11±2.642	4.28±2.346	0.01

^1^Significance associated with Independent t-test for continuous variables or Pearson’s chi-square for categorical variables

^2^Mean±SD

^3^n (%).

### Household characteristics

Household characteristics of the study participants are summarized in Table 2. The majority of women lived in extended family compounds and most of homes had mud walls and floors and aluminum roofing. Firewood was the main source of fuel for cooking. Women in forest communities spent significantly less time fetching firewood compared to in non-forest communities. Most households used solar lanterns for light at night, with higher use in the forest villages. More of the households in the non-forest area used open defecation, compared to forest areas. The main source of drinking water was from streams or rivers and only two percent of the households treated their water before drinking. Significantly more women from the non-forest villages owned animals than women in the forest villages.

**Table 2:** Household characteristics of study participants.

Characteristic	Total (N = 247)	Forest (n = 126)	Non-forest (n = 121)	^1^P-value
**Household size**	8.18±3.77^2^	8.75±4.31	7.62±3.24	0.02
**Number of rooms**	3.86±1.75	3.85±1.89	3.88±1.61	0.27
**Living arrangement**				
Extended family compound	128 (51.8)^3^	60 (47.6)	68 (56.2)	0.27
Owned single family dwelling	118 (47.8)	65 (51.6)	53 (43.8)	
Rented compound	1 (0.8)	1 (0.4)	0 (0)	
**Type of house**				
Mud	230 (93.1)	111 (88.1)	119 (98.3)	0.01
Other^4^	17 (6.8)	15 (11.9)	2 (1.7)	
**Type of roof**				
Aluminum	141 (57.1)	61 (48.4)	80 (66.1)	0.01
Thatch	106 (42.9)	65 (51.6)	41 (33.9)	
**Type of floor**				
Mud	229 (92.7)	111 (88.1)	118 (97.5)	0.01
Cement	18 (7.3)	15 (11.9)	3 (2.5)	
**Type of bed**				
Mattress	150 (60.7)	82 (65.1)	68 (56.2)	0.15
Mat	97 (39.3)	44 (35)	53 (43.8)	
**Type of fuel used in cooking**				
Firewood	245 (99.2)	125 (99.2)	120 (99.2)	1.00
Kerosene	1 (0.8)	1 (0.8)	1 (0.8)	
**Distance to fuel source (minutes)**	87.86±59.25	66.67±47.04	109.05±71.55	<0.01
**Source of light**				
Solar lanterns	165 (66.8)	107 (84.9)	58 (47.9)	<0.01
Torch	52 (20.8)	4 (3.2)	48 (39.7)	
Kerosene lanterns	30 (12.1)	15 (11.9)	15 (12.4)	
**Type of toilet facility**				
Private pit latrine	68 (27.5)	53 (42.1)	15 (12.4)	<0.01
Compound pit latrine	84 (30.4)	48 (38.1)	36 (29.8)	
Bush	95 (38.5)	25 (19.8)	70 (57.9)	
**Source of drinking water**				
River or streams	247 (100)	126 (100)	121 (100)	0.33
**Treatment of drinking water**				
No Treatment	242 (98.0)	125 (99.2)	117 (96.7)	
Treatment(boil/filter)	5 (2.0)	1 (0.8)	4 (3.3)	0.34
**Animal ownership**				
Yes	217 (87.9)	101 (80.2)	116 (95.9)	<0.01

^1^Significance associated with Independent t-test for continuous variables or Pearson’s chi-square for categorical variables

^2^Mean±SD

^3^n (%)

^4^Other; refers to cement and thatch

### Dietary intake

**Fig 3:**
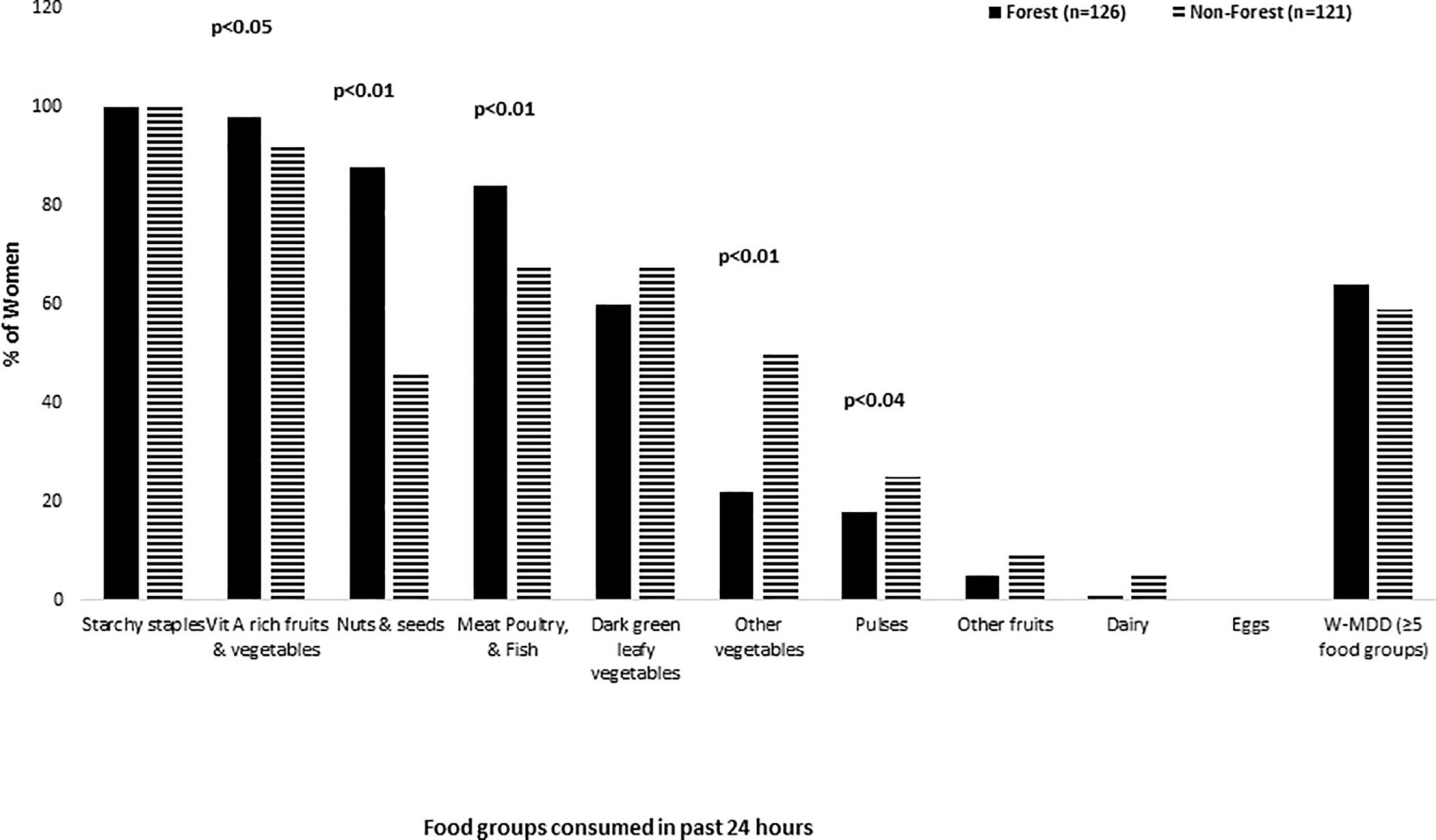
Food groups consumed by women in the past 24 hours.

The percentage of participants consuming foods from different food groups in the 24 hours preceding the survey is summarized in Fig 3. All the women in both areas had consumed foods made with starchy staples such as grains, roots and tubers or plantains in the 24-hour period captured by the survey. Compared to women from grassland villages, women from the forest-based villages were more likely to have consumed vitamin A rich fruits and vegetables (98% vs. 92%; p = 0.04), nuts and seeds (88% vs. 46%; p<0.01), and meat and fish (84% vs. 68%; p<0.01). Nuts and seeds from the forest included bush mango (*Irvingia garbonensis*, *irvingia wumbolo*) and Njansang (*Ricinodendron heudelotii*). Compared to women from forest-based villages, women from the non-forest villages were more likely to have consumed ‘other’ vegetables e.g. tomato and okra (50% vs. 22%; p<0.01), and pulses (29% vs. 18%; p = 0.03). Most of the women (61%) met the minimum dietary diversity score of ≥5 food groups. There was no difference in dietary diversity score between forest and non-forest women.

### Nutritional status of respondents

The prevalence of anemia (including mild, moderate or severe forms) among women in the study was 75.3% (Table 3). This is considered a significant public health problem based on WHO cutoffs. This prevalence rate is significantly higher than that reported in the 2011 DHS for Cameroon (53.6% for the Southwest region) [32]. The mean hemoglobin concentration for all women in the study was 10.89 ± 1.54 g/dL. Compared to women from forest villages the women from the non-forest villages had a lower mean hemoglobin concentration (11.10 ± 1.53 g/dL vs. 10.68 ± 1.55 g/dL; p = 0.03). Women from the non-forest villages had significantly higher prevalence of moderate and severe anemia, compared to women from the forest villages (73% non-forest vs 63% forest; P = 0.04). The majority of women had a normal BMI (71.3%): about 3.0% were underweight, 22.3% were overweight and 3.6% were obese.

**Table 3:** Nutritional status.

Characteristic	Total (N = 247)	Forest (n = 126)	Non-forest (n = 121)	^1^p-value
**Hemoglobin (g/dl)**	10.89±1.54^2^	11.10±1.53	10.68±1.55	0.033
**Anemia classification**				
Mild	60 (32.3)^3^	34 (37.4)	26 (27.4)	0.04
Moderate-Severe	196 (67.7)	57 (62.6)	69 (72.6)	
**Presence of Anemia**				
Any Anemia	186 (75.3)	91 (72.2)	95 (78.5)	0.252
No anemia	61 (24.7)	35 (27.8)	26 (21.5)	
**BMI**	24.5 (13.1)	23.5 (4.8)	25.5 (17.9)	
Underweight	7 (2.8)	2 (1.6)	5 (4.1)	0.428
Normal	176 (71.3)	93 (73.8)	83 (68.6)	
Overweight	55 (22.3)	28 (22.2)	27 (22.3)	
Obese	9 (3.6)	3 (2.42)	6 (5.0)	

^1^Significance associated with Independent T-test for continuous variables or Pearson’s chi-square for categorical variables

^2^Hb adjusted for altitude, Mean ± SD

^3^n (%).

### Determinants of hemoglobin levels and anemia status

Results of the regression models, presented in Table 4, confirmed that women living in the forest communities had statistically significant higher levels of adjusted hemoglobin concentration than those living in non-forest sites and lower rates of moderate to severe anemia. We ran models including several potential confounders to see what impact the addition of these had on the ‘forest’ coefficient. If the addition of the variables reduces the size and/or statistical significance of the coefficient, then this implies that it is a likely pathway through which living near forests affects anemia.

**Table 4:** Regression results (coefficient with t-statistics in parentheses).

	Adjusted hemoglobin	Moderate to Severe Anemia^$^
Forest	0.43 ** (2.19)	-0.30* (-1.85)
Constant	10.69*** (75.93)	0.18 (1.54)
Observations	247	247
R^2^	.02	
Prob>chi2		0.06

^$^ These results are for a Probit regression.

* statistically significant at 90% confidence level

** statistically significant at 95% confidence level

*** statistically significant at 99% confidence level.

Results for the multivariate regression models are reported in Table 5. The results from Model I show that differences in physiological and demographic characteristics between the two regions were not responsible for the differences in adjusted hemoglobin rates. After controlling for these, the size of the coefficient on the dummy ‘forest’ actually increased. Pregnancy status was the only physiological variable significantly associated with hemoglobin levels. Results from Model II suggest that either differences in infection are not the cause of the differences in adjusted hemoglobin or that our proxy measures for infection are not able to capture these differences adequately. None of the variables that we added to try to control for household and socio-economic characteristics in Model III were statistically significant although adding them to the model slightly reduced the importance of the forest dummy variable.

**Table 5:** Ordinary least square regression results with adjusted hemoglobin as dependent variable.

	I. Biological & demographic variables	II. Infection	III. Socio-econ characteristics	IV. Food groups	V. usual foods + forest Foods	VI Combo
Forest	0.48** (2.04)	0.48** (2.21)	0.46** (2.05)	0.64** (2.23)	0.49 (1.29)	0.67 (1.41)
Age	-0.05 (-0.39)					-0.06 (-0.37)
Age squared	0 (0.47)					0.001 (0.53)
education	-0.5 (-0.43)					-0.009 -0.06
pregnant	0.56* (1.69)					0.72 (1.59)
menstruating	-0.13 (-0.54)					-0.12 -0.41
Worms		-0.10 (-0.42)				-.17 (-0.53)
Malaria		-0.10 (-0.46)				-0.0002 (-0.00)
Household size			-0.02 (-0.74)			-0.02 (-0.48)
Wealth index			-0.05 (-0.56)			-0.002 (-0.02)
Roof material			0.20 (0.95)			0.11 (0.46)
Toilet			-0.12 (-0.54)			0.21 (0.74)
Cereals				-0.61 (-1.27)	-0.73 (-1.51)	-0.92* (-1.82)
Tuber				0.03 (0.04)	0.23 (0.25)	0.05 (0.05)
Orange colored fruit				-0.12 (-0.13)	0.20 (0.22)	0.10 (0.11)
Other fruit				-0.03 (-0.07)	-0.09 (-0.22)	-0.13 (-0.29)
Dark leafy green veg				0.45 (0.99)	0.26 (0.57)	0.65 (1.30)
Other vegetables				0.16 (0.24)	0.12 (0.17)	0.14 (0.20)
Meat				-0.61 (-1.57)	-0.67 (-1.11)	-0.51 (-0.80)
Dairy				0.48 (0.40)	0.36 (0.30)	0.51 (0.40)
Fish and seafood				-0.23 (-0.52)	-0.31 (-0.67)	-0.23 (-0.27)
Pulses				0.48 (0.83)	0.53 (0.91)	0.49 (0.82)
Seeds & Nuts				0.11 (0.16)	0.22 (0.32)	0.24 (0.34)
Oils and Fats				0.16 (0.16)	0.15 (0.15)	0.19 (0.19)
Bushmeat					0.06 (0.06)	-0.04 (-0.03)
Eru					1.41** (2.39)	1.50** (2.42)
Njansang					-0.45 (-0.63)	-0.40 (-0.51)
bushmango					-0.10 (-0.19)	-0.25 (-0.43)
constant	10.55*** (4.90)	10.99*** (21.8)	11.03*** (20.61)	10.44*** (17.90)	10.45*** (17.96)	9.83*** (3.46)
observations	247	247	247	189	189	189
R^2^	.04	.02	.03	.06	.09	.13

* statistically significant at 90% confidence level

** statistically significant at 95% confidence level

*** statistically significant at 99% confidence level.

While none of the individual food groups were statistically significant in Model IV, adding all of them to the model increases the size of the coefficient on the forest dummy by about half and it remained statistically significant. When we added controls for specific forest foods in Model V, the frequency of consumption of eru (*Gnetum africanum*), a green leaf which grows on vines in the forest, had a positive statistically significant effect on adjusted hemoglobin levels. The addition of these forest foods to the model reduced the forest dummy coefficient and made it no longer statistically significant. This implies that a likely explanation for the differences in adjusted hemoglobin between the two areas, is due to the differences in the amounts of ‘forest foods’ that women in the forest areas consume. Finally, when we put all of the independent variables together in one regression, the ‘forest’ dummy was not statistically significant and only consumption of eru had a statistically significant positive impact on adjusted hemoglobin levels. We re-ran both Models V and VI without including eru consumption and found that in both models, ‘forest’ regained its statistical significance (data not shown, available upon request).

## Discussion

The present study aimed at investigating the relationship between forest cover, dietary intake and anemia prevalence among women of reproductive ages. The nutritional status of a woman has important implications, not only for her health, but also for that of her children [2, 3]. Anemia in women of reproductive age is one of the most pressing and challenging global public health problems, and an impediment to development in developing countries, including Cameroon. Anemia is known to be especially prevalent in women living in poor and rural areas [3–5]. Studies have shown that iron deficiency may occur where there is low animal source food intake, when the majority of calories / energy in the diet comes from staple foods and especially when people are exposed to infections associated with blood loss or destruction of red blood cells [37]. The results of this study demonstrate that forests, and specifically forest foods may be protective against anemia. While several studies have previously demonstrated the importance of wild foods and forests for diet quality, this is one of the first studies to demonstrate a relationship between forest proximity, forest foods and biochemical measures of nutrition [22, 38–41].

Golden et al. [42] previously demonstrated the importance of wild animal source foods (bushmeat) for anemia. Golden et al. findings are not surprising given that diets in rural Africa usually derive a large portion of energy from carbohydrates and are low in diversity and low in animal source foods which are an excellent source of bio-available iron. Fa *et al*. found that wild meat is the primary source of animal food for most rural populations in Central Africa [43, 44]. Communities in places away from protected areas and with lower amounts of wildlife have been found to have higher rates of stunting [21]. Women in the forest areas in the current study ate animal source foods more frequently than those in the grassland area with a larger proportion of these foods coming from bushmeat. Despite this fact, consumption of meat was not statistically significant in the regression models and did not change the significance of the ‘forest’ dummy. Given that red meat is a source of bioavailable iron it was surprising that meat and bushmeat was not significantly associated with women’s hemoglobin concentration in this study. A study by Remis and Jost Robinson [45] studied hemoglobin levels in two BaAka hunter-gatherer communities adjacent to a protected area in the Central African Republic. The villages differed in the amount of bushmeat consumed (48.7% vs. 20.5% had consumed meat the day before the survey) but did not find a statistically significant different in hemoglobin levels between the villages [45]. In our study, the lack of relationship between bushmeat consumption and hemoglobin levels may be explained by the relatively high consumption of meat across all participants, with 68% of women in the non-forest communities having consumed fish, meat or poultry the previous day or by seasonal differences not measured in this study (Fig 3).

Importantly, the results of this study suggest that plant foods from the forest may also make important contributions to iron intake and reduce the risk of anemia. Several studies have shown that wild and forest foods (both plant and animal) can make important contributions to diet and nutrient intake [38–41, 46]. In Tanzania, Powell et al. [47] report almost 20% of the iron intake in the study site came from wild foods, almost all of which were wild vegetables. In a study from Benin, almost 5% of iron in the diet came from wild plant foods [40]. A review of the literature on wild foods suggested that many rural African communities obtain 60–80% of their vegetables from the wild [38], a figure that corresponds with the findings of this study. In this study site, eru (*Gnetum africanum*) is one of the most commonly consumed dark leafy vegetables and one of the most common side dishes [28]. *Gnetum africanum* is a liana that grows in the forest and is traditionally cooked with palm oil, crayfish and bird pepper.

There is a wide range of iron levels reported in the literature for *Gnetum africanum*. Differences can be due to the variety, the soil, climate and weather conditions of the place it grows, the preparation method, and the laboratory methods used to determine content. One estimate of iron content per fresh leaf (5.0 mg/100g) [48], is about 85% higher than the iron content of fresh spinach (USDA), and suggests *Eru* has relatively low levels of anti-nutrients (phytate and oxalate). The factors contributing to anemia are complex, with many interactions between various foods, nutrients, genetics and infection. It is not only the iron content of the diet that matters. For example, vitamin A enhances erythrocyte production, boosts immunity and mobilizes iron stores from tissues, thus reducing the anemia of infection [49]. Vitamin A supplementation improved the mean hemoglobin of Moroccan children by 7 g/L (p = 0.02) [50]. Eru is relatively rich in beta-carotene (a precursor to vitamin A) with about 3400 μg/100g [51] (seven times the amount found in spinach, USDA database). Moreover, it is usually prepared with vitamin-A rich palm oil. Alozeia and Ene-Obong [51] found that one common recipe for eru soup provides a 100% of vitamin A (or its precursors) for an adult. In addition, eru leaf has also been found to have anti-inflammatory properties [52], making it possible that its association with lower anemia may be due to its medicinal as opposed to dietary characteristics [53].

With respect to socio-economic (SES) factors, studies often report a positive association between wealth (or SES) and hemoglobin and/or lower rates of anemia [54]. Other studies have not found a relationship between SES and hemoglobin status [55]. Ag Ayoya et al. [56], did not find such a relationship that in West and Central Africa, in alinement with our results here There is no strong evidence of an association between education and anemia in the literature. Nwizu et al. [57] found that low educational attainment was positively associated with anemia in a sample of pregnant women in northern Nigeria, but Mekonnen et al [58] found a negative association between education and anemia among pregnant women in a hospital in Ethiopia. Thus, the results that we find here do not seem unusual.

### Limitations of the study

There are several ways that this study could be improved upon in the future. Both malaria and intestinal worms are known to contribute to lower hemoglobin levels, and we were not able to screen for these infections before measuring hemoglobin levels. Instead, we relied on self-reported medical histories which are much less accurate. Similarly, we relied on self-reports of the sickle-cell trait and sickle cell disease.

Additionally, this study would have been improved by more explicit control for exposure to environmental factors that are shaped by landscape structure, including: forest access (in terms of both distance and tenure), other vector-borne disease and other spatially-dependent factors such as exposure to human fecal matter and open defecation.

A potentially problematic issue for understanding causal pathways from forests to anemia, but one that is very difficult to control for, is the overlap between ethnicity and forest/non-forest residence. In this site, the ethnic groups that live in forest areas are different than those that live in the grasslands. In some contexts, this could mean significantly different diet, culture and habits; however, in this site ethnic groups have intermarried and have a similar culture with regards to food, taboos and livelihoods. Despite this, we cannot say for sure if the differences we observed in consumption are because of forest proximity, cultural preferences or even genetic differences in relation to anemia.

## Conclusion

Our study showed a very high prevalence of anemia—75.3%—among women around the Takamanda National Park. Women from forest communities had a higher mean hemoglobin concentration and a lower prevalence of moderate to severe anemia than women living farther from forests. In regression models, consumption of *Gnetum africanum* (eru, a common leafy vegetable from the forest) was the only variable to explain the differences in anemia between women from forest and non-forest communities. Although we have speculated on some possible reasons for these results, additional research to understand the pathways through which consumption of this plant affects hemoglobin is needed.

These results suggest that any development, agricultural or conservation intervention that leads to the loss of forests or the loss of access to forest resources for communities, may have negative implications for women’s health. This is of critical importance on its own, but because a woman’s anemia status also impacts the long-term health outcomes of her children, it could potentially have implications for the transmission of malnutrition and poverty from one generation to the next. Development strategies must find ways to improve income and reduce poverty without jeopardizing dietary quality and health of forest communities.

## Supporting information

S1 Questionnaire (DOCX)

S1 Dataset (XLSX)
