# Supplementary material for: Dietary intake, forest foods, and anemia in Southwest Cameroon
Source: PLoS One. 2019 Apr 12;14(4):e0215281. doi: 10.1371/journal.pone.0215281 (PMC6461351; doi:10.1371/journal.pone.0215281)
Supplement: S1 Questionnaire — (DOCX) [file pone.0215281.s001.docx]

**DEPARTMENT OF NUTRITON AND FOOD SCIENCE, UNIVERISTY OF GHANA**

**ASSESSMENT OF IRON STATUS IN WOMEN**

**I**nterviewer name: ___________________ Interviewer code: ___________________Interview date:__________

Participant ID: ______________ Community name: __________ Community type: 1=Forest 2= Non forest

Distance of community from nearest forest cover: ________ km

| *I would like to start by asking you some personal questions about yourself.* | | | | | |
| --- | --- | --- | --- | --- | --- |
| **Socio-demographic Characteristics** | | | | | **Data entry** |
| 01 | How old were you on your last birthday? | Age ________ years | | |  |
| 02 | What is your ethnic background? | 1=Anyang  2=Becheve  3=Bayangi  4=Other (specify) | | |  |
| 03 | How long have you lived in this village? | ________Years | | |  |
| 04 | What is your religious affiliation? | 1= Christian  2= Muslim  3= Traditional belief  4= Other (specify) | | |  |
| 05 | What is the highest level of formal education you have completed? | 1=None  2=Nursery  3=Primary  4=Secondary  5=Tertiary | | |  |
| 06 | What is your primary source of income | 1=Farmer  2=Trader  3=Seamstress  4=Hair dresser  5=Other (Specify)  6=None | | |  |
| 07 | What is your current marital status? | 1=Single (never been married)  2=Married  3=Divorced  4=Cohabiting  5=Widowed | | |  |
| 08 | What does your spouse/partner do for a living? | 1=Farmer  2=Trader  3=Other (Specify)  99=Not applicable | | |  |
| 09 | What is his highest level of formal education? | 1=None  2=Nursery  3=Primary  4=Secondary  5=Tertiary  99= Not applicable | | |  |
| 10 | How many adults and children are in your household (or eat from the same pot)? | No. of adults (≥18 yrs) _______ | | |  |
|  |  | No. of children (< 18 yrs) _______ | | |  |
|  |  | Total household size _______ | | |  |
| 11 | Is any member of your household engaged in any forest-based income generating activity? (tick all that apply) |  | **YES=1** | **NO=2** |  |
|  |  | Self |  |  |  |
|  |  | Spouse/partner |  |  |  |
|  |  | Children |  |  |  |
|  |  | Parent |  |  |  |
|  |  | In-law |  |  |  |
|  |  | Other, specify |  |  |  |
| 12 | Which forest derived income generating activities are you or members of your households involved in? (Tick) |  | **YES=1** | **NO=2** |  |
|  |  | Bushmeat |  |  |  |
|  |  | Timber |  |  |  |
|  |  | Firewood/Charcoal |  |  |  |
|  |  | Fruits |  |  |  |
|  |  | Vegetables |  |  |  |
|  |  | Rattan |  |  |  |
|  |  | Medicinal herbs |  |  |  |
|  |  | Seeds/nuts |  |  |  |
|  |  | Other, specify |  |  |  |
|  |  | 99=Not Applicable |  |  |  |
| 13 | In your opinion, about how much of your HH income is contributed by the forest-based income generation activities | 1= up to 25%;  2= 26-50%;  3= 51-75%;  4=76-100%;  5=None | | |  |
| 14 | Have you ever been part of a savings association or club? | 1=Yes in the past  2=Yes presently  3=No, never | | |  |
| *Now I will ask you questions about your household* | | | | | |

| **Household Characteristics** | | | | | | |  |
| --- | --- | --- | --- | --- | --- | --- | --- |
| 15 | | | What is your current living arrangement | 1= Extended family compound setting  2= Rented compound setting  3=Rented Single family dwelling  4= Owned single family dwelling  5= Other(please specify) | | |  |
| 16 | | | How many rooms does your home have? |  | | |  |
| 17 | | | What is the main type of material for the walls (if this can be observed then no need to actually ask the question) | 1= Cement  2= Mud  3= Wood  4= Thatch  5= Other (specify) | | |  |
| 18 | | | What is the material of most of the roof? | 1= Aluminium sheets  2= Thatch  3= Other (specify) | | |  |
| 19 | | | What type of material is the floor made of? | 1=cement  2=mud  3=other (specify)_____________ | | |  |
| 20 | | | What type of bed is most commonly used for sleeping? | 1=Mattress; 2= Mat; 3= Other (Specify) | | |  |
| 21 | | | What kind of toilet facility does your household use? | 1= Private pit latrine; 2= Compound pit latrine; 3= Bush; 4= Other (specify) | | |  |
| 22 | | | What is your main source of drinking water? | 1= Pipe; 2= Well; 3= River/stream; 4= Other | | |  |
| 23 | | | How far is your home from the nearest water source? | 1= Within the community  2= Outside the community (>200m) | | |  |
| 24 | | | How do you treat the water before using for drinking? | 1=No treatment;  2= Boil, 3= Filter,  4=Sedimentation,  5=Other, specify ______________ | | |  |
| 25 | | | What is your main source of light? | 1= Electricity,  2=Solar lamp,  3= kerosene lamp,  4= Other, specify______________ | | |  |
| 26 | | | What fuel does your household mostly use in cooking? | 1=Firewood  2=Kerosene stove  3=gas  4=charcoal  5=Other, specify ______________ | | |  |
| 27 | | | Distance from source of fuel (minutes of walking) |  | | |  |
| 28 | | | Does your household own any of the following in working condition? |  | **YES=1 NO=2** | |  |
|  |  |  |  | Radio |  | |  |
|  |  |  |  | Phone |  | |  |
|  |  |  |  | Bicycle |  | |  |
|  |  |  |  | TV |  | |  |
|  |  |  |  | Motorcycle |  | |  |
|  |  |  |  | Chainsaw |  | |  |
|  |  |  |  | Generator |  | |  |
|  |  |  |  | Car |  | |  |
|  |  |  |  | Other items worth ≥25,000frs  __________________ |  | |  |
|  |  |  |  | Other items worth ≥25,000frs |  | |  |
| 29 | | | Does your household rear any of the following animals? |  | **1=YES NO=2** | |  |
|  |  |  |  | Chicken |  | |  |
|  |  |  |  | Ducks |  | |  |
|  |  |  |  | Goat |  | |  |
|  |  |  |  | Sheep |  | |  |
|  |  |  |  | Pigs |  | |  |
|  |  |  |  | Cattle |  | |  |
|  |  |  |  | Other specify___________ |  | |  |
|  |  |  |  | Other specify___________ |  | |  |
| *Now I will ask you questions about your health* | | | | | | | |
| **Reproductive History** | | | | | | | |
| 30 | | Have you ever been pregnant? | | 1=Yes; 2=No | | |  |
| 31 | | If yes, how many live births have you had? | |  | | |  |
| 32 | | Are you currently pregnant? | | 1=Yes; 2=No | | |  |
| 33 | | Are you currently having your menses? | | 1=Yes; 2=No | | |  |
| **HEALTH AND FOREST USE QUESTIONS** | | | | | | | |
| 34 | Do you have sickle cell trait? | | | 1=Yes; 2=No; 3=don’t know | | |  |
| 35 | Have you had Malaria in the last two weeks? | | | 1=Yes; 2=No (***If No skip to Q37***) | | |  |
| 36 | When you have malaria which of the following do you do to treat it? | | |  | | **YES=1 NO=2** |  |
|  |  |  |  | Go to the health post | |  |  |
|  |  |  |  | Buy medicine from pharmacy/store | |  |  |
|  |  |  |  | Use medicine from forest | |  |  |
|  |  |  |  | Other specify ____________ | |  |  |
|  |  |  |  | Other, specify ____________ | |  |  |
| 37 | Have you had any problems with stomach worms in the last month? | | | 1=Yes; 2=No | | |  |
| 38 | How do you treat it? | | |  | | **YES=1 NO=2** |  |
|  |  |  |  | Go to the health post | |  |  |
|  |  |  |  | Buy medicine from pharmacy/store | |  |  |
|  |  |  |  | Use medicine from forest | |  |  |
|  |  |  |  | Other specify ____________ | |  |  |
|  |  |  |  | Other, specify ____________ | |  |  |
| 39 | Have you taken iron supplements in the last 6 months? | | | 1=Yes; 2=No | | |  |
| 40 | Have you experienced blood shortage in the last 6 months? | | | 1=Yes; 2=No | | |  |
| 41 | How do you treat it? | | |  | | | |
| 42 | What do you do to prevent malaria? | | |  | | **YES=1 NO=2** |  |
|  |  |  |  | Use a mosquito net | |  |  |
|  |  |  |  | Medication | |  |  |
|  |  |  |  | Forest based medication | |  |  |
|  |  |  |  | Other, specify | |  |  |
|  |  |  |  | NONE | |  |  |
| 43 | What do you do to prevent worm infestation? | | |  | | **YES=1 NO=2** |  |
|  |  |  |  | Regular deworming | |  |  |
|  |  |  |  | Drink treated water | |  |  |
|  |  |  |  | Forest based medicine | |  |  |
|  |  |  |  | Other, specify | |  |  |
|  |  |  |  | NONE | |  |  |
| 44 | Could you please tick the forest foods that you ate in the last week | | |  | | **YES=1 NO=2** |  |
|  |  |  |  | Eru | |  |  |
|  |  |  |  | Bush Mango | |  |  |
|  |  |  |  | Njansang | |  |  |
|  |  |  |  | Contri Onion | |  |  |
|  |  |  |  | Bush Pepper | |  |  |
|  |  |  |  | Okongobong | |  |  |
|  |  |  |  | Others (Please Specify) | |  |  |
|  |  |  |  |  |  |  |  |
|  |  |  |  |  |  |  |  |
|  |  |  |  |  |  |  |  |

**DIETARY DIVERSITY QUESTIONNAIRE**

**Please describe the foods (meals and snacks) that you ate or drank yesterday during the day and night, whether at home or outside the home. Start with the first food or drink of the morning.**

*Write down all foods and drinks mentioned. When composite dishes are mentioned, ask for the list of ingredients. When the respondent has finished, probe for meals and snacks not mentioned.*

| Approx. time of eating event | Meal/Snack | Actual foods eaten | Quantity | Recipes | Source of food: 1=forest, 2= farm, 3 = purchase, 4= other, specify |
| --- | --- | --- | --- | --- | --- |
|  |  |  |  |  |  |
|  |  |  |  |  |  |
|  |  |  |  |  |  |
|  |  |  |  |  |  |
|  |  |  |  |  |  |

Write down the SOURCE of each of these foods next to it, e.g. (wild/forest, farm, purchase, other)

*When the respondent recall is complete, fill in the food groups based on the information recorded above. For any food groups not mentioned, ask the respondent if a food item from this group was consumed*

| **SN** | **Food group** | **Examples** | **Sources: 1=Forest,2=farm,**  **3=purchase,4=other(sp)** |
| --- | --- | --- | --- |
| 1 | **CEREALS** |  |  |
|  |  |  |  |
|  |  |  |  |
| 2 | **WHITE ROOTS AND TUBERS** |  |  |
|  |  |  |  |
| 3 | **VITAMIN A RICH VEGETABLES AND TUBERS** |  |  |
|  |  |  |  |
|  |  |  |  |
| 4 | **DARK GREEN LEAFY VEGETABLES** |  |  |
|  |  |  |  |
|  |  |  |  |
| 5 | **VITAMIN A RICH FRUITS** |  |  |
|  |  |  |  |
|  |  |  |  |
|  |  |  |  |
| 6 | **OTHER VEGETABLES** |  |  |
|  |  |  |  |
| 7 | **OTHER FRUITS** |  |  |
|  |  |  |  |
| 8 | **ORGAN MEAT** |  |  |
|  |  |  |  |
| 9 | **FLESH MEATS** |  |  |
|  |  |  |  |
| 10 | **EGGS** |  |  |
|  |  |  |  |
| 11 | **FISH AND SEAFOODS** |  |  |
| 12 | **LEGUMES NUTS AND SEEDS** |  |  |
|  |  |  |  |
|  |  |  |  |
| 13 | **MILK AND MILK PRODUCTS** |  |  |
| 14 | **OILS AND FATS** |  |  |
|  |  |  |  |
| 15 | **SPICES, CONDITMENTS, BEVERAGES** |  |  |
|  |  |  |  |
| 16 | **BUSHMEAT, CATERPILLARS, INSECTS** |  |  |
|  |  |  |  |
| 17 | **RED PALM PRODUCTS** |  |  |

i. Did you eat anything (meal or snack) OUTSIDE the home yesterday? 1=Yes; 2=No _____________

ii. Was your food intake typical or unusual yesterday? ___________

iii. If unusual, please explain.

**ANTHROPOMETRY and HB FORM**

Name of interviewer___________________________________________________________________________

Date of interview_______________________ Location (FBV/GBV) _______________________

Woman’s ID

| **Measurement** | **Weight (Kg)** | **Height (cm)** | **Hb (g/dL)** |
| --- | --- | --- | --- |
| Measurement 1 |  |  |  |
| Measurement 2 |  |  |  |
| Average |  |  |  |
